# A participatory study on the knowledge, attitudes, and practices of poultry farmers regarding vaccine use in the northern region of Bangladesh

**DOI:** 10.5455/javar.2025.l944

**Published:** 2025-08-18

**Authors:** Md. Sodrul Islam, Apurbo Kumar Mondal, Md. Rabiul Auwul, Md. Shahidul Islam, Kazi Khalid Ibne Khalil, Md. Mizanur Rahman, Obaidul Islam, A.K.M. Ziaul Haque, Jahid Hasan Tipu, Md. Altafur Rahman, Md. Ashraful Islam, Md. Aminul Islam, Mohammad Shah Alam

**Affiliations:** 1 Department of Physiology and Pharmacology, Gazipur Agricultural University, Gazipur, Bangladesh; 2 Department of Agricultural and Applied Statistics, Gazipur Agricultural University, Gazipur, Bangladesh; 3 Laboratory of Veterinary Epidemiology, College of Veterinary Medicine, Chungbuk National University, Cheongju-si, South Korea; 4 Kazi Farms Poultry Laboratory, Gazipur, Bangladesh; 5 Department of Clinical Science, Faculty of Medicine, University of Bergen, 5020 Bergen, Norway; 6 Laboratory of Animal Genetics, Graduate School of Innovation and Practice for Smart Society, Hiroshima University, Hiroshima, Japan; 7 Department of Livestock Services (DLS), Ministry of Fisheries and Livestock (MOFL), Dhaka, Bangladesh; 8 Department of Medicine, Gazipur Agricultural University, Gazipur, Bangladesh; 9 Department of Anatomy and Histology, Gazipur Agricultural University, Gazipur, Bangladesh

**Keywords:** Knowledge, attitudes, and practices (KAP), poultry farmers, Bangladesh, vaccine use

## Abstract

**Objective:**

The study aimed to assess poultry farmers‘ (PF) knowledge, attitudes, and practices (KAP) about the utilization of vaccines for the prevention of infectious illnesses.

**Materials and Methods:**

A cross-sectional investigation was carried out involving 260 respondents in the northern area of Bangladesh. Data were collected by structured questionnaires with randomly selected participants. The analysis used descriptive statistics and logistic regression.

**Results:**

Most respondents were male (81.5%), aged 31–40 years (32.3%), with secondary education (27.7%), as well as vaccination training (30.8%). Although 63.1% of participants were aware of immunizations, only 41.5% recognized they prevented zoonotic infections, and 66.9% reduced antibiotic use. Remarkably, 67.7% knew about the bad effects, and 70.8% said they are vaccinating their chicken flocks. Overall, 41.5%, 48.5%, and 29.2% of the farmers demonstrated good knowledge and a positive attitude, as well as performed better practices. Multivariable analyses found that male farmers aged over 50 years with 3–5 years of broiler farming expertise and having undergone vaccination training demonstrated a higher likelihood of possessing substantial knowledge regarding vaccine utilization. Accordingly, favorable attitudes were connected with male farmers aged over 50 years and having 3–5 years of broiler farming experience. Farmers who engaged in broiler farming demonstrated a higher likelihood of exhibiting effective vaccination practices only.

**Conclusion:**

The study highlights gaps in farmers‘ KAP related to vaccine usage. It is essential to create targeted educational as well as training programs to effectively address these gaps and prevent possible poultry illnesses.

## Introduction

Bangladesh‘s economy relies heavily on the agriculture sector, particularly livestock, which contributes 1.85% to the overall Gross Domestic Product (GDP), having a share of 16.52% of agricultural GDP in the 2022–23 fiscal year. The Department of Livestock Services in Bangladesh reported that livestock covers a large population of 442.847 million, including 385.704 million chickens, with poultry providing 37% of the total animal protein source [1,2]. The poultry industry in this country is on the rise to supply sufficient egg and meat products, mainly following two types: backyard and commercial. Commercial production includes broilers, layers, and Sonali chickens, a hybrid breed for meat and egg production [3]. Commercial poultry farming has a significant role in increasing the country’s revenue and employment opportunities for a large number of people to ensure financial prosperity, but the main impediment is the occurrence of diseases [4]. A study identified 25 different avian illnesses and disease conditions in Bangladesh, hindering the industry‘s expansion [5].

Infections caused by bacteria, viruses, parasites, and fungi significantly affect the quality and quantity of poultry products. Viral diseases such as avian influenza, infectious bronchitis, Newcastle disease, and infectious bursal disease led to productivity declines, posing challenges to the poultry industry‘s expansion [6]. Vaccination is crucial to prevent disease spread, with various approaches implemented at international, national, and farm levels [6]. Vaccines play a vital role in minimizing outbreaks and fostering poultry production growth by enhancing immunity against specific infections. Overall, vaccination is the most effective strategy for managing infectious illnesses in chickens, preventing disease through enhanced immunity with biologically generated antigens [6].

To optimize the advantages of chicken farming, timely vaccination administration is crucial for effective disease management in smallholder or larger-scale [7]. However, whether psychological or economic aspects affecting households‘ vaccination choices are unexplored up to this time [8,9]. Infectious diseases may cause detrimental effects on health in underdeveloped countries [10]. Poultry vaccines safeguard consumers, boost chicken productivity, and lead to improved returns in comparison to investment by preventing mortality and improving internal health status [7]. Due to increasing concerns regarding antimicrobial resistance, vaccination is essential for disease management to protect the lives of animals and humans [11]. Proper livestock vaccination approaches should be made to ensure that harmful chemical- or drug-residue-free meat, eggs, and milk for consumption [7]. However, research on vaccine efficacy across various livestock species in Bangladesh is lacking.

Livestock producers‘ knowledge, attitudes, and practices (KAP) are critical for proper vaccination approaches, confirming better vaccine efficacy as well as sustainability [12]. Vaccination attitudes are impacted by factors such as cost, accessibility, and cultural views [9]. A regional study revealed that in Ethiopia, vaccination rates were lower due to farmers‘ limited knowledge regarding disease occurrence and the usefulness of immunization [13].

Moreover, farmers had a limited understanding of vaccine storage, handling, and delivery protocols in another region of that country [14]. The degree to which livestock producers adhere to recommended vaccination schedules and procedures varies, according to studies on their immunization habits [14,15]. To increase effective immunization coverage and decrease unsuccessful vaccinations, more vaccine management training is essential.

Poultry producers often face financial challenges that hamper their ability to get vaccines. As a result, poultry farms have low vaccination coverage and insufficient knowledge about the importance of vaccinations [10,16]. Therefore, the development of effective vaccination methods requires a thorough understanding of farmers‘ previous knowledge, opinions, and behaviors around vaccine use [12,17]. Therefore, the study was intended as novel work to assess the significance of KAP among the poultry producers of Bangladesh regarding vaccination against viral diseases to find the limitations and necessary measures to be taken to make a better scenario in this field. The findings of the study may be regarded as the background information for preparing effective vaccination guidelines for disease prevention and control.

## Materials and Methods

### Ethical statement

The Animal Research Ethics Committee (AREC) of Gazipur Agricultural University granted approval for the research protocol (FVMAS/AREC/2023/7), following a comprehensive evaluation. Each participant gave their informed consent, which ensured that they were able to participate voluntarily and that their rights and personal details were kept private.

### Research location and duration of the study

**Figure 1.**
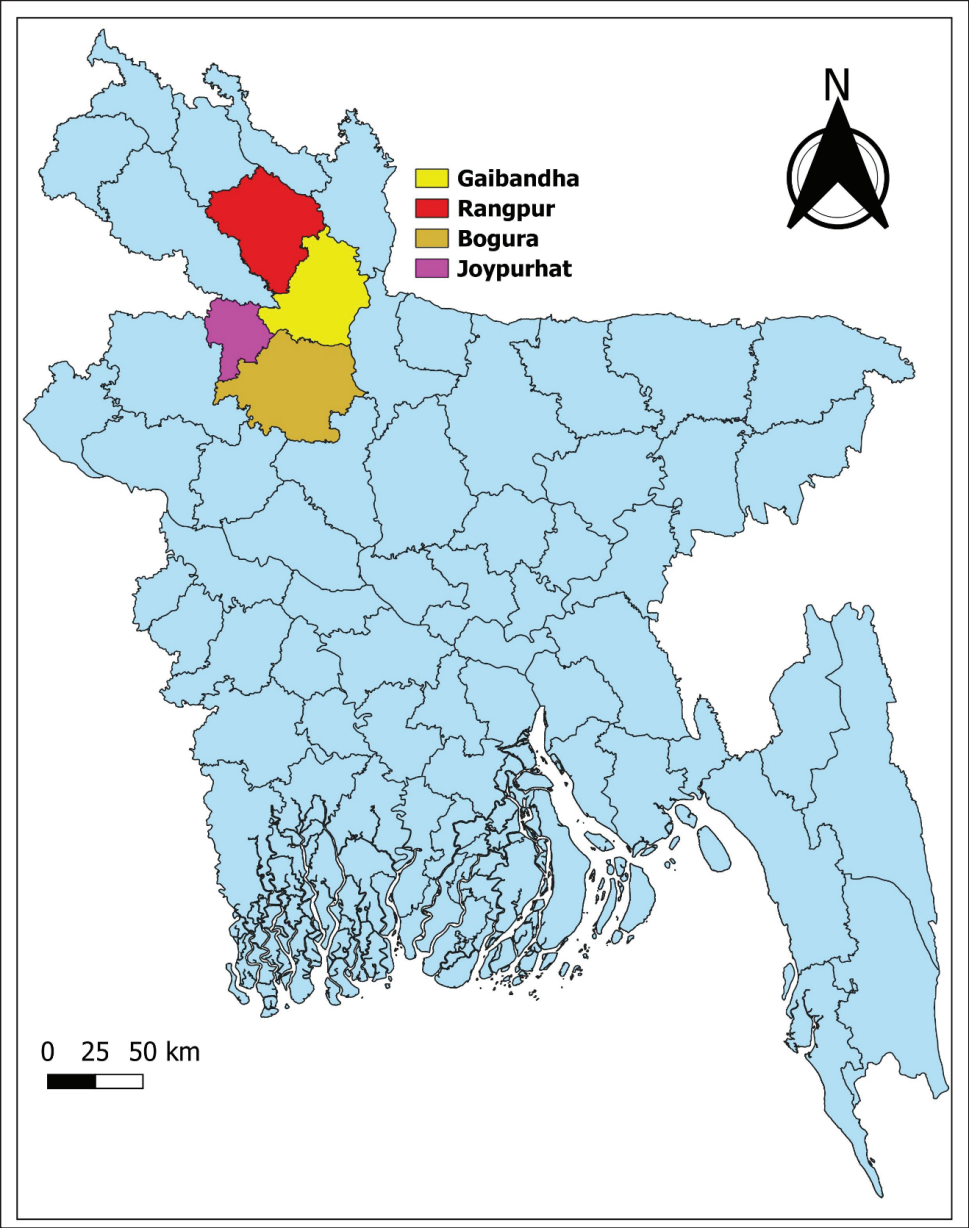
The map of the survey area in Bangladesh visually, with different colors showing polls in particular districts.

The study was carried out in four districts in the northern part of Bangladesh: Rangpur, Gaibandha, Bogura, and Joypurhat (Fig. 1). A comprehensive survey was conducted over 16 upazilas, with four chosen from every district for analysis. The research was implemented from July to December 2023.

### Research plan and methods for sampling

The present KAP research was conducted using a cross-sectional survey. Data were collected from 260 farmers, comprising 80 layer farmers, 80 Sonali farmers, and 100 broiler farmers. Farmers were chosen at first from a list given through the Upazila Livestock Offices; their involvement in the survey was dependent upon their willingness. To make sure the sample was genuinely representative and random; participants were then chosen at random from the group. The Raosoft model volume computation method was used to establish the sample size for our research [18]. A 50% response rate distribution with a 95% confidence level (CI) and a 5% margin of error was used. A nonresponse rate of 5% was also considered [18]. A 50% sample percentage was chosen because of the dearth of equivalent studies for this cohort in the specified study location. As a result, 196 was the minimal sample size required for our evaluation. To ensure the strength of the study, 260 participants in total were gathered.

### Development of surveys and data collection

The study employed a questionnaire that comprised four parts (A to D). Section A collected demographic data, including age, gender, education, district, farm type, period of farming expertise, and vaccine-associated training. Sections B and C focused on knowledge and attitude, respectively, each containing 13 unique closed-ended questions (K1−K13 for knowledge as well as A1−A12 for attitudes). Section D, which assessed practices, included 12 questions (P1−P12), comprising both closed-ended and open-ended formats. The questionnaire underwent pre-testing with a sample of 20 poultry farmers (PF) and was subsequently revised based on the findings. Data collection was performed by veterinarians along with veterinary students via in-person interviews employing questionnaires based on papers.

### Data management, scoring, and statistical analysis

The information was recorded in an MS Excel file for purification and then analysis. A scoring system was used to assess the participants‘ KAP levels; correct responses received a score of 1, whereas incorrect responses received a score of 0. The correct responses for every question were compiled to calculate an overall score for each KAP domain. The maximum achievable scores were 13 for knowledge, along with attitude, as well as 12 for practice. Every participant’s overall score for every KAP domain was subtracted from the highest conceivable score for that domain, and the result was multiplied by 100 to determine their percentage score. A cut-off criterion of 60% was applied to measure degrees of good knowledge as well as practice, and the data were then divided into two groups according to the ratio of correct answers to KAP-level inquiries. Participants achieving scores over 60% were categorized as possessing favorable views, whereas those scoring below this level were considered to have poor knowledge, negative attitudes, and poor practices [19]. To further analyze the interrelationship among the KAP scores, Spearman‘s rank correlation coefficient was applied.

The statistical study was conducted applying SPSS version 26 from IBM Corp. Descriptive statistics were employed to assess categorical variables, including frequency and percentage. We employed both univariate and multivariate analyses to examine the connections among independent factors (sociodemographic) as well as dependent variables (KAP) at a significance level of *p* < 0.05. The univariable logistic regression method was employed to determine the odds ratio (OR) as well as the 95% CI for different sociodemographic factors. Subsequent to the assessment procedure, only univariable factors with *p* < 0.20 were merged into the final multivariate analysis [20].

Additionally, we utilized the backward elimination method to do a multivariate logistic regression analysis. The adjusted ORs (AORs), as well as 95% CIs, were then determined using the final multivariate logistic regression analysis. A *p*-value below 0.05 was measured as statistically significant, with results reported as 95% CIs and AOR. Statistical significance was evaluated at a *p*-value threshold of less than 0.05, with results described as AOR and 95% CIs. The model‘s overall fit in KAP techniques was evaluated through Hosmer−Lemeshow goodness-of-fit evaluations [21].

## Results

### Sociodemographic characteristics of PF

In the northern parts of Bangladesh, we carried out a study that was a cross-sectional investigation. The participants consisted of 260 PF from four different districts: Rangpur, Gaibandha, Bogura, and Joypurhat. The majority of the 260 PF were male (81.5%), spanning various age groups, with significant representation in the 31–40 years group (32.3%) and the over 50 years group (20.8%). Participants had diverse educational backgrounds, with 18.5% lacking formal education and 12.3% holding graduate or higher degrees. The study area was divided into two regions: Rangpur and Gaibandha (26.9%) and Bogura and Joypurhat (23.1%). Broiler farms comprised 38.4%, layer farms 30.8%, and Sonali farms 30.8%. Although there were differences in farm experience, most (34.6%) had 6–10 years of experience. Nevertheless, knowledge was deficient as well as protocols concerning vaccines, since only 30.8% had received training on their use (Table 1).

### Farmers‘ knowledge of vaccine use

The results indicate that most (63.1%) of PF are familiar with vaccines. Approximately 40% of PF (39.6%) possess knowledge about illnesses affecting poultry, and 43.1% are aware that their farms have a prior history of diseases. Regarding vaccine belief, 55.4% of PF perceive them as effective, but merely 18.5% recognize priority immunizations. Furthermore, a notable percentage (61.5%) doubt the effectiveness of vaccines in preventing uncommon diseases, and 40.8% question the need for non-vaccine illness preventive strategies.

Notably, 67.7% of respondents are concerned about possible side effects, and 37.7% think that some vaccines are superior to others. Just 31.5% of respondents recognize the advantages of vaccinations, and only 41.5% recognize the significance of immunizations in halting the spread of zoonotic diseases. A notable proportion (63.1%) expresses skepticism concerning the efficiency of routine immunizations in mitigating antibiotic resistance, while 36.9% possess doubts about this capability. Furthermore, 53.8% acknowledge that certain major poultry diseases can solely be controlled via vaccination. Significant differences (*p* < 0.05) in knowledge about vaccine use were found among the PF, except for the K4 and K13 variables (Table 2).

### Farmers‘ attitudes toward vaccine use

A considerable proportion of PF concurs with the accessibility of vaccinations for avian diseases (55.4%) and the notion that a single vaccination provides permanent immunity (46.2%), while disagreeing with the notion of equal protection against all diseases (52.3%). In contrast, 37.7% of PF concur that all flocks should be safeguarded, even if certain chickens are unvaccinated, reflecting a moderate comprehension of flock immunity principles. Most PF (53.8%) believe that vaccines are more costly than alternative illness prevention strategies. However, there is widespread agreement on the importance of vaccines for improving productivity and well-being (57.7%), the need for highly effective vaccines (63.5%), and their role in reducing antibiotic use (66.9%). Furthermore, a considerable number of individuals believe that vaccinated poultry are less susceptible to illness (64.6%) and that vaccines are typically considered safe for both people and poultry (71.5%).

**Table 1.** Sociodemographic features of PF (*n =* 260) in the survey region.

Variables	Category	Frequency (Number)	Percentage
Gender	Male	212	81.5%
	Female	48	18.5%
Age	18–30 years	46	17.7%
	31–40 years	84	32.3%
	41–50 years	76	29.2%
	≥ 50 years	54	20.8%
Education	No formal education	48	18.5%
	Primary	52	20.0%
	Secondary	72	27.7%
	Higher secondary	56	21.5%
	Graduation and above	32	12.3%
District	Rangpur	70	26.9%
	Gaibandha	70	26.9%
	Bogura	60	23.1%
	Joypurhat	60	23.1%
Type of farm	Broiler	100	38.4%
	Layer	80	30.8%
	Sonali	80	30.8%
Experience in farming	≤ 2 years	46	17.7%
	3–5 years	84	32.3%
	6–10 years	90	34.6%
	> 10 years	40	15.4%
Training in livestock illnesses and immunization	Received	80	30.8%
	Not received	180	69.2%

Although there are differences in views, most people (45.8%) think that government financing for vaccines is a good idea, while 61.5% agree that vaccines help ensure food safety. Additionally, 58.5% of PF concur that immunizations have an impact on sustainable chicken production. Similar to knowledge, attitudes toward vaccine use varied significantly among PF, except for A1, A2, A4, and A6 variables (*p* < 0.05) (Table 3).

**Table 2.** Evaluation of participants‘ knowledge about vaccine utilization for avian illnesses.

Factors	Categories	Frequency (*n =* 260)	Proportion (%)	*p*-value
K1. Have you been informed about vaccines for poultry?				
	Yes	164	63.1%	0.000
	No	96	36.9%	
K2. Knowledge of poultry diseases				
	Yes	103	39.6%	0.001
	No	157	60.4%	
K3. History of prior diseases on the farm				
	Yes	112	43.1%	0.026
	No	148	56.9%	
K4. Are poultry vaccines capable of effectively preventing diseases in chickens?				
	Yes	144	55.4%	0.082
	No	116	44.6%	
K5. Understanding of priority poultry vaccinations				
	Yes	48	18.5%	0.000
	No	212	81.5%	
K6. Poultry vaccines protect against rare illnesses that do not impact your chickens.				
	Yes	160	61.5%	0.000
	No	100	38.5%	
K7. Should poultry illnesses be limited and stopped without vaccinations?				
	Yes	106	40.8%	0.003
	No	154	59.2%	
K8. Some poultry vaccines exhibit higher efficacy than others				
	Yes	98	37.7%	0.000
	No	162	63.3%	
K9. Poultry health could be negatively impacted by vaccination				
	Yes	176	67.7%	0.000
	No	84	32.3.6%	
K10. Knowing the advantages of poultry vaccination				
	Yes	82	31.5%	0.000
	No	178	68.5%	
K11. Vaccinating chickens successfully stops the spread of zoonotic diseases				
	Yes	108	41.5%	0.006
	No	152	58.5%	
K12. Can regular vaccination help to lower problems with antibiotic resistance in chicken farms?				
	Yes	96	36.9%	0.000
	No	164	63.1%	
K13. Vaccination is the only treatment for several poultry diseases				
	Yes	140	53.8%	0.215
	No	120	46.2%	

**Table 3.** Evaluation of participants‘ attitudes about vaccine utilization for avian illnesses.

Factors	Categories	Frequency (*n =* 260)	Proportion (%)	*p*-value
A1. Poultry disease vaccines are readily accessible				
	Agree	144	55.4%	0.082
	Disagree	92	35.4%	
	Neutral	24	9.2%	
A2. Do you believe one vaccine can provide lifelong immunity to chickens?				
	Agree	120	46.2%	0.215
	Disagree	92	35.4%	
	Neutral	48	18.5%	
A3. Should a single vaccine offer uniform safeguards against all avian illnesses?				
	Agree	92	35.4%	0.000
	Disagree	136	52.3%	
	Neutral	32	12.3%	
A4. Poultry vaccines are costlier than alternative disease prevention measures				
	Agree	140	53.8%	0.215
	Disagree	81	31.2%	
	Neutral	39	15.0%	
A5. If some birds in a flock are vaccinated and others are not, should all flocks be protected?				
	Agree	98	37.7%	0.000
	Disagree	126	48.5%	
	Neutral	36	13.8%	
A6. The government needs to provide funds for poultry immunizations				
	Agree	119	45.8%	0.172
	Disagree	94	36.2%	
	Neutral	47	18.1%	
A7. Vaccinating poultry can lower the necessity for antibiotics in chicken				
	Agree	174	66.9%	0.000
	Disagree	65	25.0%	
	Neutral	21	8.1%	
A8. Poultry vaccination is required to increase chicken welfare and productivity				
	Agree	150	57.7%	0.000
	Disagree	92	35.4%	
	Neutral	18	6.9%	
A9. A vaccination with high efficacy is crucial				
	Agree	165	63.5%	0.000
	Disagree	71	27.3%	
	Neutral	24	9.2%	
A10. Vaccinated healthy chickens have a lower risk of illness				
	Agree	168	64.6%	0.000
	Disagree	70	26.9%	
	Neutral	22	8.5%	
A11. Vaccines are widely utilized in people and animals due to their safety profile				
	Agree	186	71.5%	0.000
	Disagree	50	19.2%	
	Neutral	24	9.2%	
A12. Vaccinating farm chicken enhances the safety of our food supply				
	Agree	160	61.5%	0.000
	Disagree	68	26.2%	
	Neutral	32	12.3%	
A13.Vaccination helps to make poultry husbandry more sustainable				
	Agree	152	58.5%	0.006
	Disagree	80	30.8%	
	Neutral	28	10.8%	

### Practices of farmers regarding vaccine use

Two-thirds of the PF (70.8%) reported vaccinating their poultry flocks, primarily based on vaccination date and time (34.2%). However, a significant portion (60.8%) of producers do not maintain immunization records or adhere to a regular immunization regimen (62.7%). A significant portion (25.4%) of respondents indicated that vaccines for specific diseases were unavailable. Furthermore, a majority of PF (58.8%) depend on veterinarian prescriptions for vaccine purchases and engage in reviewing the vaccine brochure (54.6%). In terms of storage practices, the majority (52.7%) of PFs store their food in multifunctional refrigerators. It is concerning that a significant percentage of PF (57.7%) fail to verify vaccine expiration dates, and a substantial majority (84.2%) do not properly dispose of utilized or expired vials. Additionally, a significant percentage of PF (64.6%) indicated inadequate immunization practices as well as a record of vaccine failure (23.1%). The level of vaccine use among PF varied significantly, similar to the variation in knowledge, except for P2 and P7 variables (*p* < 0.05) (Table 4).

### Factors influencing farmers‘ KAP concerning vaccine use

**Knowledge of the farmers**

**Figure 2.**
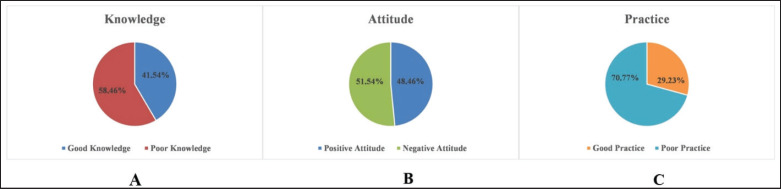
KAP of PF regarding vaccine utilization A, B, and C show the score of PF regarding vaccine utilization of knowledge, attitude and practice, respectively.

The findings of the current investigation discovered that 41.5% of respondents had strong knowledge overall (Fig. 2A). Univariable examination showed substantial relations (*p* < 0.05) among participants‘ knowledge levels as well as factors such as gender, age, education, type of farm, farming expertise, and vaccination training. Male producers exhibited 4.46-fold greater odds of having good knowledge regarding poultry vaccine usage compared to females.

**Table 4.** Evaluation of participants‘ practice about vaccine utilization for avian illnesses.

Factors	Categories	Frequency (*n =* 260)	Proportion (%)	*p*-value
P1. Do you often immunize your poultry flocks?				
	Yes	184	70.8	0.000
	No	76	29.2%	
P2. When do you administer vaccinations to your chickens?				
After the spread of illnesses		74	28.5%	0.804
Advice from fellow farmers.		54	20.8%	
Recommendation for a veterinarian		43	16.5%	
Based on the date and time of vaccination		89	34.2%	
P3. Do you maintain a record of previous poultry immunizations administered on the farm?				
	Yes	102	39.2%	0.001
	No	158	60.8%	
P4. Does your poultry farm have a regular immunization schedule?				
	Yes	97	37.3%	0.000
	No	163	62.7%	
P5. Do you have any diseases for which you are now unable to obtain a vaccine?				
	Yes	66	25.4%	0.000
	No	194	74.6%	
P6. Do you buy chicken vaccines according to a specific prescription from a veterinarian?				
	Yes	153	58.8%	0.004
	No	107	41.2%	
P7. Do you review the prospectus prior to providing poultry vaccines?				
	Yes	142	54.6%	0.137
	No	118	45.4%	
P8. Where are your vaccines stored?				
Particular refrigerator exclusively for poultry vaccine		105	40.4%	0.002
Multifunctional refrigerator		137	52.7	
Non-refrigerated cabinet		13	5.0%	
Others		5	1.9%	
P9. Do you verify the expiration dates of vaccines prior to administering them to chickens?				
	Yes	110	42.3%	0.013
	No	150	57.7%	
P10. Are you correctly discarding bottles and vials of utilized or expired chicken vaccine?				
	Yes	41	15.8%	0.000
	No	219	84.2%	
P11. Have the chickens been vaccinated appropriately?				
	Yes	92	35.4%	0.000
	No	168	64.6%	
P12. Does your poultry farm have a record of vaccine failure?				
	Yes	60	23.1%	0.000
	No	200	76.9%	

Likewise, farmers aged 41–50 demonstrated a greater proficiency in understanding the use of poultry vaccines (OR: 5.65; 95% CI: 2.39–13.34) in contrast to people in the 18–30 age range. Moreover, those with primary (OR: 6.32; 95% CI: 2.40–16.66) or secondary (OR: 5.85; 95% CI: 2.32–14.77) schooling showed noticeably greater levels of good knowledge than individuals who had no formal education. Compared to Sonali farmers, broiler farmers demonstrated noticeably elevated degrees of good knowledge (OR: 10.50; 95% CI: 4.84–22.76). Remarkably, farmers who had worked for more than 10 years performed better (OR: 4.40; 95% CI: 1.72–11.23) in terms of knowledge compared to those with ≤ 2 years of experience. Furthermore, compared to their peers who were not trained, farmers who received poultry vaccine instruction from any organization possessed greater knowledge (OR = 3.44, 95% CI: 1.88–6.28). Districts, however, did not show up in this investigation as a major determinant (Table 5). The multivariate examination indicated that knowledge of PF varied substantially by age (*p* = 0.009), gender (*p* = 0.003), type of farm (*p* = 0.000), experience in farming (*p* = 0.005), and vaccination training (*p* = 0.000) (Table 5).

**Attitude of the farmers**

The findings of the current survey exhibited a 48.5% overall positive attitude score (Fig. 2B). Univariate examination revealed substantial correlations (*p* < 0.05) among positive attitude scores as well as variables such as age, gender, educational level, type of farm, and farming experience. Male farmers exhibited a 2.41-fold greater likelihood of possessing a favorable attitude toward chicken vaccine utilization in comparison to their peers. Similarly, farmers aged 41 to 50 showed a more favorable attitude toward vaccination than those aged 18 to 29 (OR: 4.11; 95% CI: 1.86–9.07). In contrast to farmers who lack formal education, those who had finished secondary school (OR: 4.23; 95% CI: 1.91–9.35) showed noticeably greater levels of positive attitude.

Broiler farmers exhibited a notably higher level of positive attitude (OR: 3.42; 95% CI: 1.83–6.40) in contrast to the farmers of Sonali. It is interesting to note that farmers with over 10 years of experience outperformed those with less than 2 years in the attitude category (OR: 4.77; 95% CI: 1.88–12.05). The multivariate examination discovered that the attitude of PF substantially varied based on gender (*p* = 0.040), age (*p* = 0.004), type of farm (*p* = 0.000), and experience in farming (*p* = 0.002). Nevertheless, this study found no statistically significant variance in the remaining variables (Table 6).

**Practice of the farmers**

Regarding poultry vaccines, just 29.2% of the farmers who participated in this survey showed an acceptable level of practice (Fig. 2C). Substantial correlations (*p* < 0.05) were discovered by the univariate analysis between the practice level of the respondents and their age, farm category, and farming experience. Specifically, older farmers, particularly those aged 50 years and above, were significantly more likely to demonstrate good practice in poultry vaccines (OR: 5.21; 95% CI: 1.77–15.33) in contrast to farmers between the ages of 18 and 30 years.

Compared to Sonali farmers, broiler farmers had noticeably enhanced levels of effective practices (OR: 3.47; 95% CI: 1.66–7.24). It is interesting to note that farmers with over 10 years of experience outperformed those with less than 2 years in the practice arena (OR: 3.71; 95% CI: 1.33–10.33). However, this research revealed that gender, education, districts, and vaccination training were not statistically significant factors. According to a multivariate study, broiler producers outperformed Sonali farmers in terms of practice (OR: 3.16, 95% CI: 1.47–6.80). However, in this study, there was no statistically significant change in the remaining variables (Table 7).

**Table 5.** Univariable and multivariable studies showing the link between demographic factors and the degree of knowledge, *n =* 260.

Factors	Knowledge level		Univariable analyses OR (95%CI) *p*-value		Multivariable analyses Adjusted OR (95%CI) *p*-value	
	Good (%)	Poor (%)	OR (95% CI)	*p-*value	AOR (95% CI)	*p*-value
Gender						
Male	100	112	4.46 (1.99–9.99)	0.000	5.07 (1.71–15.02)	0.003
Female	8	40	Ref.		Ref.	
Age						
31–40 years	26	58	1.84 (0.77–4.36)	0.000	0.26 (0.60–1.19)	0.009
41–50 years	44	32	5.65 (2.39–13.34)		3.74 (1.12–12.53)	
≥ 50 years	29	25	4.76 (1.93–11.77)		4.08 (0.75–22.09)	
18–30 years	9	37	Ref.		Ref.	
Education						
Primary	27	25	6.32 (2.40–16.66)	0.003	4.01 (1.15–13.91)	0.111
Secondary	36	36	5.85 (2.32–14.77)		4.32 (1.37–13.59)	
Higher secondary	25	31	4.72 (1.81–12.32)		4.79 (1.31–17.47)	
Graduation and above	13	19	4.00 (1.37–11.65)		5.40 (1.28–22.71)	
No formal education	7	41	Ref.		Ref.	
District						
Rangpur	28	42	0.81 (0.40–1.63)	0.933		
Gaibandha	28	42	0.81 (0.40–1.63)			
Bogura	25	35	0.87 (0.42–1.79)			
Joypurhat	27	33	Ref.			
Farm type						
Broiler	60	40	10.50 (4.84–22.76)	< 0.000	15.40 (5.95–39.87)	< 0.000
Layer	38	42	6.33 (2.86–14.02)		9.04 (3.50–23.33)	
Sonali	10	70	Ref.		Ref.	
Experience in farming						
3–5 years	35	49	2.57 (1.12–5.86)	0.016	2.82 (0.84–9.47)	0.005
6–10 years	41	49	3.01 (1.33–6.80)		1.48 (0.43–5.08)	
> 10 years	22	18	4.40 (1.72–11.23)		1.30 (0.20–8.38)	
≤ 2 years	10	36	Ref.		Ref.	
Training in livestock illnesses and immunization						
Received	18	62	3.44 (1.88–6.28)	< 0.000	5.48 (2.45–12.22)	< 0.000
Not received	90	90	Ref.		Ref.	

CI: confidence interval; OR: odd ratio; AOR: Adjusted odd ratio; Ref: reference category.

Education on livestock illnesses and immunization.

**Relations between the KAP of farmers**

The Spearman‘s rank correlation test discovered a positive relation among the KAP scores, as indicated in Table 8. There was a substantial correlation of 0.35 (*p* < 0.001) between the scores of knowledge as well as attitude. In a similar vein, it was shown that there was a connection of 0.36 (*p* < 0.001) between the scores of knowledge as well as practice. The coefficient of association between attitude and practice was found to be the lowest overall, with an average value of 0.172 (*p* < 0.001). In addition, there was a moderately positive connection between knowledge and practice, as well as between knowledge and attitude. On the other hand, there was a minimal positive association between practice and attitude.

**Table 6.** Univariable and multivariable studies display the link between demographic variables and the degree of attitudes, *n =* 260.

Factors	Attitude level		Univariable analyses		Multivariable analyses	
	Positive (%)	Negative (%)	OR (95% CI)	*p* value	AOR (95% CI)	*p* value
Gender						
Male	111	101	2.47 (1.24–4.71)	0.009	2.47 (1.04–5.87)	0.040
Female	15	33	Ref.		Ref.	
Age						
31–40 years	43	41	2.66 (1.23–5.75)	0.008	2.11 (0.49–8.98)	0.004
41–50 years	47	29	4.11 (1.86–9.07)		7.22 (2.40–21.67)	
≥ 50 years	23	31	1.88 (0.81–4.35)		2.68 (0.62–11.54)	
18–30 years	13	33	Ref.		Ref.	
Education						
Primary	24	28	2.30 (0.99–5.33)	0.011	1.68 (0.62–4.54)	0.070
Secondary	44	28	4.23 (1.91–9.35)		3.12 (1.27–7.65)	
Higher secondary	28	28	2.69 (1.18–6.14)		2.16 (0.80–5.83)	
Graduation and above	17	15	3.05 (1.19–7.82)		3.71 (1.21–11.29)	
No formal education	13	35	Ref.		Ref.	
District						
Rangpur	32	38	0.84 (0.42–1.68)	0.905		
Gaibandha	33	37	0.89 (0.44–1.77)			
Bogura	31	29	1.06 (0.52–2.18)			
Joypurhat	30	30	Ref.			
Farm type						
Broiler	58	42	3.42 (1.83–6.40)	0.000	3.89 (1.94–7.79)	0.000
Layer	45	35	3.18 (1.65–6.13)		3.71 (1.81–7.58)	
Sonali	23	57	Ref.		Ref.	
Experience in farming						
3–5 years	44	40	3.50 (1.57–7.80)	0.004	5.42 (2.13–13.75)	0.002
6–10 years	47	43	3.47 (1.57–7.69)		3.71 (1.58–8.72)	
> 10 years	24	16	4.77 (1.88–12.05)		4.76 (1.78–12.73)	
≤ 2 years	11	35	Ref.		Ref.	
Training in livestock illnesses and immunization						
Received	42	38	0.79 (0.46–1.34)	0.385		
Not received	84	96	Ref.			

CI: confidence interval; OR: odd ratio; AOR: Adjusted odd ratio; Ref: reference category.

## Discussion

### Knowledge of the farmers

Our initial investigation aims to evaluate the KAP of chicken producers concerning vaccine utilization in Bangladesh. We further examine characteristics that forecast the overall KAP between individuals. Our data reveal both similarities and discrepancies with previous studies on livestock vaccine-associated KAP [12,17]. According to our research, farmers‘ average level of knowledge was 41.5% (Fig. 2A), comparable to research in Oromia, Ethiopia [12], although inferior to another investigation in Southwest Ethiopia [17].

Additionally, 39.6% of farmers demonstrated moderate knowledge of poultry diseases (Table 2), consistent with earlier studies [8,15] emphasizing disease knowledge and transmission for effective vaccination. Prior studies have emphasized the importance of historical background in disease management [22,23]. Prior epidemics significantly impact farmers‘ understanding of vaccination, as our results corroborate. It is essential to prioritize illnesses for poultry immunization to achieve effective disease management [7]. Previous research in Bangladesh found that important diseases for vaccination include infectious bursal disease, Newcastle disease, Marek‘s disease, mycoplasmosis, fowl pox, fowl cholera, salmonellosis, colibacillosis, infectious bronchitis, infectious coryza, and avian influenza. Our investigation found that less than 20% of participants were knowledgeable about this priority poultry vaccination, conforming to the most recent Ethiopian study [7].

**Table 7.** Univariable and multivariable studies showing the link between demographic factors and the degree of practices, *n =* 260.

Factors	Practice level		Univariable analyses		Multivariable analyses	
	Good (%)	Poor (%)	OR (95% CI)	*p* value	AOR (95% CI)	*p* value
Gender						
Male	59	153	0.70 (0.36–1.36)	0.298		
Female	17	31	Ref.			
Age						
31–40 years	24	60	3.28 (1.15–9.30)	0.021	1.49 (0.42–5.28)	0.146
41–50 years	26	50	4.26 (1.50–12.09)		3.65 (1.13–11.77)	
≥ 50 years	21	33	5.21 (1.77–15.33)		5.05 (1.07–23.73)	
18–30 years	5	41	Ref.		Ref.	
Education						
Primary	19	33	2.87 (1.11–7.41)	0.144	2.79 (0.99–7.86)	0.325
Secondary	26	46	2.82 (1.15–6.94)		2.45 (0.94–6.36)	
Higher secondary	15	41	1.82 (0.699–4.78)		1.86 (0.640–5.41)	
Graduation and above	8	24	1.66 (0.55–5.02)		1.82 (0.53–6.18)	
No formal education	8	40	Ref.		Ref.	
District						
Rangpur	19	51	0.80 (0.37–1.71)	0.888		
Gaibandha	19	51	0.80 (0.37–1.71)			
Bogura	19	41	1.00 (0.46–2.15)			
Joypurhat	19	41	Ref.			
Farm type						
Broiler	38	62	3.47 (1.66–7.24)	0.004	3.16 (1.47–6.80)	0.011
Layer	26	54	2.72 (1.26–5.90)		2.58 (1.16–5.73)	
Sonali	12	68	Ref.		Ref.	
Experience in farming						
3–5 years	28	56	2.78 (1.10–7.06)	0.041	3.10 (0.93–10.36)	0.264
6–10 years	25	65	2.14 (0.84–5.41)		1.17 (0.39–3.55)	
> 10 years	16	24	3.71 (1.33–10.33)		1.19 (0.23–5.99)	
≤ 2 years	7	39	Ref.		Ref.	
Training in livestock illnesses and immunization						
Received	19	61	1.48 (0.81–2.71)	0.197	1.21 (0.62–2.34)	0.572
Not received	57	123	Ref.		Ref.	

CI: confidence interval; OR: odd ratio; AOR: Adjusted odd ratio; Ref: reference category.

Our result indicated that a majority of producers hold a negative view of the health benefits of poultry immunization (Table 2), which supports data from a study conducted in southwest Ethiopia [12]. This perception could change by sharing evidence-based data on the benefits of poultry vaccines, including lowering disease rates as well as enhanced chicken welfare [17, 23]. Interestingly, farmers who failed to immunize their animals did not report facing any challenges [24]. Accordingly, 40.8% of survey participants thought that diseases affecting poultry could be prevented and controlled without immunization. Instructing farmers on the aims and advantages of vaccination programs is essential to enhance comprehension and acceptance of poultry vaccines [9]. Furthermore, the majority of producers in our survey perceived vaccines as effective in avoiding chicken illnesses, consistent with recent research done in southwest Ethiopia [12]. Conversely, a large majority (61.5%) of producers in our survey thought that vaccines could efficiently safeguard their chickens from rare illnesses that do not significantly affect overall health. This result aligns with earlier research done in southwest Ethiopia [12] as well as Oromia, Ethiopia [17].

**Table 8.** Relationships among KAP and vaccine utilization (*p* ≤ 0.001).

Factors	Correlation coefficient	*p*-value
Knowledge-attitudes	0.351	0.000
Knowledge-practices	0.365	0.000
Attitudes-practices	0.172	0.000

Furthermore, vaccines are crucial in public health as they reduce the reliance on antibiotic treatments, which in turn limits the transmission of antimicrobial resistance. Furthermore, the adoption of immunization protocols also aids in preventing zoonotic diseases [25]. In our study, a considerable number of poultry producers indicated that routine immunizations might alleviate concerns regarding antibiotic resistance (Table 2). They regarded vaccination as an efficacious means of stopping the transmission of zoonotic illnesses. Furthermore, these farmers viewed immunization as the sole solution for certain illnesses.

In line with previous research [12,17], the multivariate examination in the present investigation demonstrates that farmers‘ knowledge varied significantly depending on various sociodemographic characteristics, including gender, age, experience in farming, farm type, and training (Table 5). Male participants aged more than 50 years and those who had been engaged in broiler production for 3–5 years, as well as those who had received immunization training, knew considerably more about chicken vaccines than their peers. This phenomenon may occur because as farmers grow older and gain experience in the field, they learn a lot about raising poultry and veterinary care. This enhanced knowledge provides them with a clearer understanding of the implications as well as strategies for managing poultry diseases by immunization.

### Attitude of the farmers

The average attitude score of the participants was 48.5% (Fig. 2B), consistent with similar research findings [12,17]. Increasing livestock vaccination rates could involve adjusting the disease monitoring system and improving vaccine accessibility [26]. Our findings revealed that 55.4% of participants were aware of the availability of vaccines for poultry diseases (Table 3), aligning with a previous study [12]. Additionally, participants‘ attitudes were influenced by the cost of poultry vaccines, consistent with findings from similar research [12,16,27]. Financial factors can pose obstacles to implementing poultry vaccines, emphasizing the economic considerations individuals consider [9]. Like previous investigations [12,27], our analysis revealed a relationship among participants‘ vaccine utilization as well as their perceptions toward government accountability in subsidizing poultry vaccination.

Vaccination protects the health of people as well as livestock by avoiding illness outbreaks, thereby enhancing poultry productivity [17]. Our survey demonstrated that most producers believe that vaccinations are safe for both people and animals and contribute to increased chicken production and welfare. Research demonstrates that immunizations can lessen the requirement for antibiotics in animals raised for food [28]. Most farmers in our study thought that immunizing their chicken flocks could reduce the usage of antibiotics, demonstrating their consciousness of antibiotic resistance. Poultry have a higher immunological response compared to mammals, making them more susceptible to vaccines that boost their innate immunity [29]. Our study found that less than half of the participants think a single vaccination provides lifelong immunity for chickens, in line with earlier research conducted in southwest Ethiopia [12]. This idea stands in opposition to the scientific evidence, which shows that numerous vaccines need booster doses to attain both optimal and sustained immunity [30]. Additionally, one-third (35.4%) of PF in our study think that one vaccine should offer equal protection against all illnesses, consistent with similar research findings [12]. Nevertheless, our study participants‘ views of the feasibility as well as the effectiveness of immunization programs were shaped by their recognition of the need for many vaccines to protect chickens from different diseases [28]. Vaccination assistance programs have demonstrated efficacy in decreasing avian mortality, hence improving food security as well as raising egg intake among mothers and young kids [31]. Most of the producers in our survey believe that chicken immunizations contribute to safer food, indicating a level of public health awareness (Table 3). The participants‘ attitudes were notably connected to the individual vaccination of chickens within a flock for comprehensive protection. Increasing livestock vaccination is a crucial strategy for meeting the United Nations Sustainable Development Goals [15]. Our investigation revealed that some farmers perceive vaccination as enhancing the sustainability of poultry farming.

Like previous research [12,17], the multivariate examination in the present investigation demonstrates that farmers‘ attitudes varied significantly depending on various sociodemographic characteristics, including gender, age, type of farm, and farming experience (Table 6). Male participants aged more than 50 years who had been engaged in broiler production for 3–5 years had considerably more favorable attitudes regarding poultry vaccines than their peers. The levels of attitude were closely aligned with knowledge levels, indicating a significant positive correlation between the two. This correlation can also be ascribed to the possibility that participants‘ attitudes were influenced by their level of knowledge, which is consistent with prior studies [12,17]. Surprisingly, training does not necessarily lead to positive attitudes, despite its typical enhancement of knowledge levels.

### Practice of the farmers

Our research indicated that producers were inadequately converting their knowledge as well as attitudes into practice. The average practice result was 29.2%, which is inferior to the knowledge score (41.5%) as well as the attitude score (48.5%) (Fig. 2). Despite this, most farmers (70.8%) in our study reported vaccinating their chicken flocks, less than previous investigations conducted in Bangladesh [32], yet greater than findings from a study in southwest Ethiopia [12]. Additionally, a noteworthy correlation was discovered between the date of chicken vaccines and the immunization practices of the respondents. The study also indicated that a large number of farmers seek guidance from veterinarians, while others get help from other producers or wait until diseases spread (Table 4). The disparities in opinions could result from variances in how effective vaccines are thought to be, personal experience, or levels of trust in veterinary skills [12]. Additionally, our survey found that 39.2% of farmers keep records of vaccinations, consistent with findings from similar studies [20,21,27].

However, our survey found that only 37.3% of poultry producers adhered to vaccination schedules to prevent diseases, which is a lower practice rate than reported in a recent study conducted in Bangladesh (79%) [33]. This inconsistent practice has the potential to cause disease epidemics anywhere, as well as at any time. Although vaccination is the most effective method for avoiding most poultry diseases, a shortage of suitable vaccines has hampered its widespread application [9]. The present study found that one-fourth (25.4%) of respondents indicated the unavailability of specific vaccines, a figure that exceeds findings from previous studies [9,16]. Compared to a study conducted in Bangladesh, less than half (45.4%) of respondents engaged in inappropriate practices by failing to read the prospectus on the vaccination bottle [21]. Before administering a vaccination, it is advised to verify the vial‘s expiration date and discard those that have passed [6]. However, the majority of producers in our survey failed to verify the expiration dates of vaccines or to appropriately dispose of eliminated or expired vials. Proper immunization is vital for keeping a farm free of disease. Despite this, our findings show that only one-third (35.4%) of producers have properly immunized their chickens, a lower rate than reported in Bangladesh [16]. In South Africa, smallholder livestock producers encounter difficulties with vaccine storage. They frequently store vaccines in the same refrigerator as food, which creates the possibility of food contamination as well as unintentional consumption by children. As a result, 31% of producers in South Africa declined to use refrigerated vaccines due to security concerns, whereas 19% expressed uncertainty [27]. However, it is alarming that half (52.7%) of farmers utilized a multipurpose refrigerator to store vaccines alongside food items (Table 4). Thus, implementing extensive training as well as awareness initiatives for these farmers is crucial to mitigate any risks associated with such practices.

A prior study suggested that farm type substantially affects knowledge, attitudes, and management practices related to poultry vaccines [12]. The multivariate analysis results in the study show that farm type is the only sociodemographic variable significantly affecting farmers‘ vaccination practice scores (Table 7). This finding aligns with a study [23], which found that farm type affects livestock vaccination knowledge, attitudes, and implementation. However, education did not significantly influence the practice score. This result is unexpected, as prior studies suggested that improved practice was associated with higher levels of education [12,20]. This gap may result from inadequate practice levels identified in our study if appropriate strategies and awareness are lacking.

### Limitations of the study

This study presents several limitations associated with collecting human behavior data through surveys. We initially conducted personal interviews to administer the KAP questionnaire. However, some farmers may have provided socially desirable answers, potentially affecting data accuracy. Participants self-reported their attitudes and past activities, which could result in inaccuracies due to poor recall and confirmation bias. Second, the sample consisted of a limited number of participants from each of the four districts in Bangladesh‘s northern area. The limited sample size might not sufficiently reflect the KAP of poultry producers nationwide. Furthermore, the study did not differentiate KAP levels among rural, urban, and peri-urban regions, potentially affecting the results. We recommend incorporating this demographic aspect into future research. Lastly, KAP survey methods may inadvertently prompt participants to provide responses they believe are acceptable or favorable to the researcher. This cross-sectional approach could affect the understanding of the causal relationship among predictor factors as well as the dependent binary elements (KAP) among poultry producers.

## Conclusion

Our study provides the first comprehensive evaluation of KAP regarding vaccine utilization among PF in Bangladesh. The investigation findings suggest that 41.5% of them possess good knowledge, 45.5% maintain a favorable attitude, while only 29.2% follow proper immunization practices. These results emphasize a significant gap in the successful application of KAP among farmers. Furthermore, we determined that farmers‘ KAP about vaccine usage is substantially influenced by sociodemographic factors, including gender, age, type of farm, expertise in farming, and training. However, we found that educational status did not significantly influence the results. Therefore, specific interventions are required to boost farmers‘ KAP in this region. Recommended strategies include educational training programs that increase awareness and encourage the adoption of more effective vaccination practices.

## List of abbreviations

AOR, adjusted odds ratios; AREC, The Animal Research Ethics Committee; CI, confidence interval; GDP, gross domestic product; KAP, Knowledge, attitudes, and practices; OR, odds ratio; PF, poultry farmers.
